# Uric acid and cardiometabolic diseases

**DOI:** 10.1186/s40885-020-00146-y

**Published:** 2020-06-15

**Authors:** Seung Jae Lee, Byeong Kil Oh, Ki-Chul Sung

**Affiliations:** Division of Cardiology, Department of Internal Medicine, Kangbuk Samsung Hospital, Sungkyunkwan University School of Medicine, 29 Saemunan-ro, Jongno-gu, Seoul, 03181 Republic of Korea

**Keywords:** Uric acid, Cardiometabolic disease, Cardiovascular disease, Metabolic syndrome

## Abstract

Hyperuricemia, which has been considered as a cause of gout and nephrolithiasis has recently been suggested to be associated with hypertension, coronary heart disease, heart failure, atrial fibrillation, insulin resistance, and nonalcoholic fatty liver disease. Several clinical and experimental studies have supported uric acid (UA) as an independent risk factor for predicting disease development along with the traditional risk factors. The mechanism by which UA causes cardiometabolic disease has not been fully elucidated to date; however, it has been explained by several hypotheses such as oxidative stress, reduced nitric oxide bioavailability, inflammation, endothelial dysfunction, and so on. Although evidence of the preventive and therapeutic effects of UA lowering therapy on cardiometabolic diseases is still insufficient, it is expected to be considered as a new treatment strategy for such diseases through additional, carefully designed, large-scale clinical studies.

## Introduction

Uric acid (UA) is the end product of purine metabolism in humans, it is formed from xanthine by xanthine oxidase enzyme through several steps and excreted into the urine. UA is endogenously synthesized mainly in the liver, intestines, muscle, and vascular endothelium [1, 2]. Exogenously, UA can be increased by intake of red meat, seafood, fatty food, alcohol, sugar-sweetened (fructose) drinks, and so on [3–5]. In addition, UA levels are increased in a state of rapid cell turnover such as tumor lysis syndrome, leukemia, lymphoma, or myeloproliferative disease. Hyperuricemia treatment can be divided into two main categories, namely, reducing UA production with xanthine oxidase inhibitors (febuxostat, allopurinol, etc.), and increasing UA excretion by using uricosurics (probenecid, benzbromarone, etc.).

In many mammals, UA is converted to highly soluble allantoin and maintained at very low levels (approximately 1 mg/dL; 60 μmol/L) [6]. Meanwhile, because the urate oxidase or uricase gene is modified to an unexpressed (pseudogene) state in humans, UA is no longer catabolized to allantoin and becomes the end product of purine metabolism. Eventually, UA is maintained at the theoretical limit of solubility in the serum (6.8 mg/dL), and is periodically excreted in the urine, mostly supersaturated [7].

Pathologically, increased serum UA levels lead to crystal (monosodium urate [MSU]) precipitation in the joints, soft tissue, kidneys, and other organs, which in turn causes various diseases [8, 9]. It has been known for decades that UA has a significant role in gout and kidney stones formation [7, 10]. Gout, a crystalline arthropathy, has become increasingly common in the last few decades [11]. Its prevalence among US adults is 3.9%, and hyperuricemia prevalence, which is a prerequisite for gout development, is 14.6% [12]. Furthermore, gout prevalence is 0.76% in Korea, 1.1% in China, 2.49% in the UK, and 0.9% in France [13–16].

The role of UA in CVD or cardiometabolic disease is still controversial because it is perceived that UA plays a protective role in oxidative stress. Otherwise speaking, UA acts as an active oxygen scavenger in the human body and has an antioxidant effect that prevents cardiovascular diseases (CVD), such as atherosclerosis [17, 18]. In support of this, a study of healthy volunteers showed that the antioxidant effect of UA was substantially greater than that of the ascorbic acid [19, 20].

**Fig. 1:**
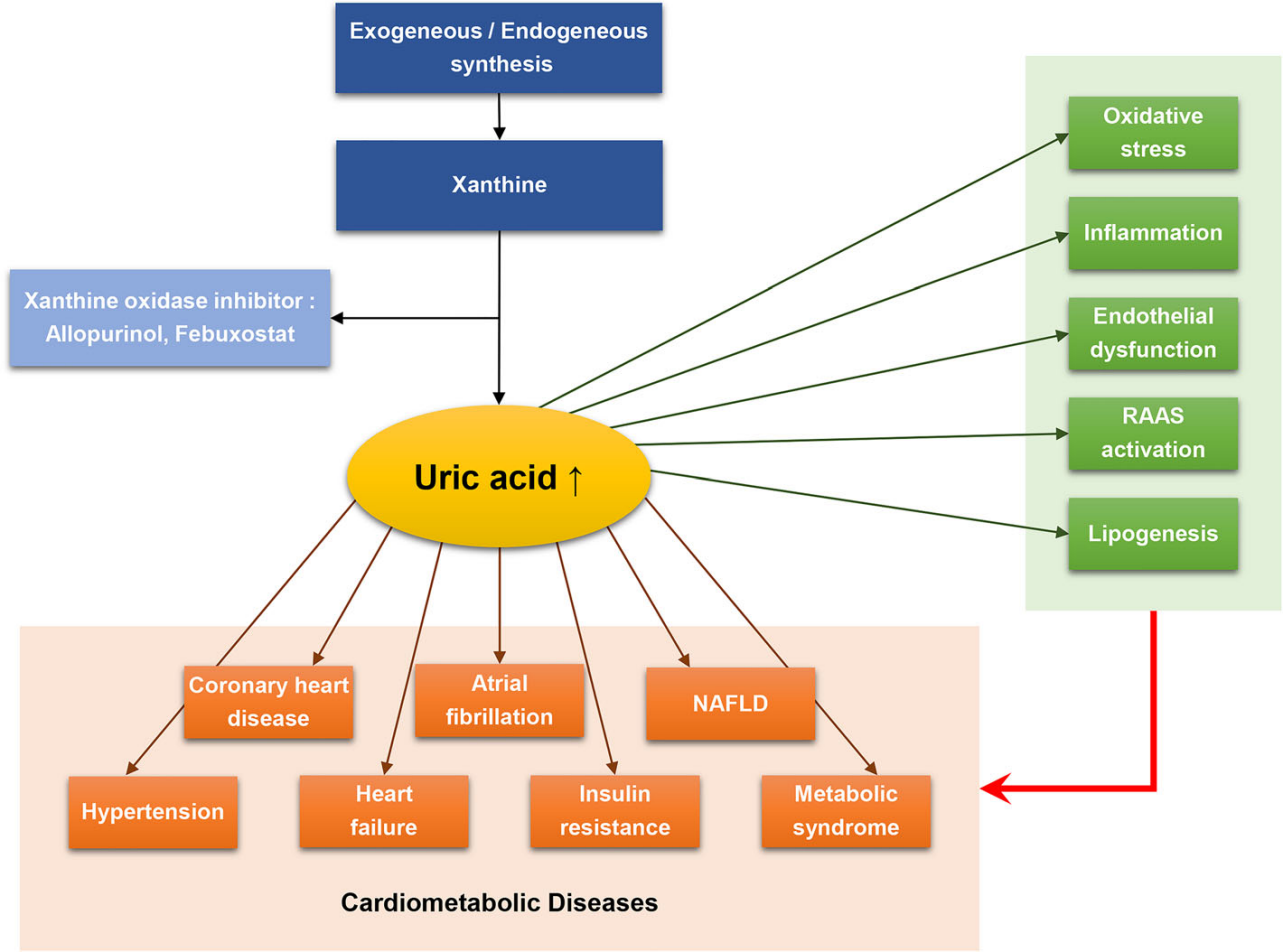
Schematic diagram showing interplay of uric acid, metabolic syndrome and CVD. CVD, cardiovascular disease; RAS, renin-angiotensin-aldosterone system; NAFLD, Non-alcoholic fatty liver disease

However, several clinical and epidemiological studies have presented the relationship between UA and various disorders including CVD, metabolic syndrome, and kidney disease, thereby overwhelming the beneficial effects of UA [21–25] (Fig. 1). Furthermore, hyperuricemia can be associated with cardiometabolic diseases as an independent risk factor in asymptomatic subjects without comorbidities [26]. In this review, we discuss the relationship between UA and cardiometabolic disease.

## Uric acid and hypertension

Several studies have demonstrated that hyperuricemia is associated with hypertension development [26–28]. A recent meta-analysis study reported that the risk of incident hypertension increased by 13% for every 1 mg/dL increase in the UA level [27]. The linear relationship between hypertension and UA levels was valid despite the UA levels being within the normal range, and in the absence of a threshold [29]. We postulated that asymptomatic hyperuricemia in the healthy population demonstrated to be an independent risk factor for the occurrence of incident hypertension [30]. The effect of UA on incident hypertension was more remarkable in younger individuals and women [31]. However, the relationship between UA and hypertension was not related to racial differences [26, 32].

In experimental studies, it was suggested that UA inhibits nitric oxide release from the endothelial cells, activates the renin-angiotensin system, and increases oxidative stress, which damages the endothelial cells and causes vasoconstriction, leading to hypertension development [33–37]. In addition, genetic variations in *SLC2A9* and *GLUT9,* associated with the regulation of UA levels in the human body, are known to be associated with hypertension development [38–41].

## Uric acid and coronary heart disease

CVD is the leading cause of death worldwide, with ischemic heart disease accounting for nearly 50% of all CVD mortalities, according to the World Health Organization (WHO) estimates [42]. Since the past decades, clinical and experimental studies have suggested that UA was associated with CVD. Furthermore, it was argued that UA could be considered as a potential therapeutic target, proving that it is an independent risk factor for CVD [43, 44]. In an observational cohort study of 5115 recruited young adult participants, hyperuricemia was shown as an independent risk factor for subclinical atherosclerosis [44]. In another large cohort study, the Rotterdam study, 4385 adults aged 55 years and older showed that the increased UA levels were independent prognostic factors of cardiovascular events and all-cause mortality [45]. A meta-analysis study reported that for every 1 mg/dL increase in the serum UA level, the overall coronary heart disease (CHD) risk increased by 20% and all-cause mortality increased by 9% [46]. In other studies, the UA levels and both all-cause and cardiovascular mortalities had a U-shape relationship [47]. Furthermore, the association between UA and CVD was found to be stronger in women than in men [48]. Several cohort and meta-analysis studies of individuals with coronary artery disease have also shown that UA increases mortality [49–51]. A meta-analysis of nine studies with 25,229 patients with confirmed or suspected CHD suggested that every 1 mg/dL increase in the UA level was associated with a 12 and 20% increase in the cardiovascular and all-cause mortalities, respectively [51]. Several experimental studies have further suggested that UA causes CHD through the mechanism of reducing nitric oxide in the endothelial cells, inhibiting endothelial proliferation, and inducing platelet adhesiveness as well as activating proliferative and inflammatory pathways in the vascular smooth muscle [52–54]. In addition, it has been hypothesized that UA causes endothelial dysfunction by increasing oxidative stress through xanthine oxidase, thus affecting CHD [55]. Allopurinol, a xanthine oxidase inhibitor, reduced the risk of myocardial infarction (MI); however, colchicine did not support this hypothesis [56].

On the contrary, some studies including ours, have argued that serum UA did not meaningfully improve the prediction of CHD in the general population and was not associated with all-cause and cardiovascular mortalities [57, 58]. Furthermore, two recent Mendelian randomization studies did not demonstrate a clear causal relationship between UA and CHD [59, 60]. Such evidence suggests that further research will need to be carefully conducted, taking into account all possible confounding factors, in order to reach a clear conclusion about the association and causality between UA and CHD.

## Uric acid and heart failure

Hyperuricemia is frequently found in heart failure patients, and UA levels are elevated in more than half of the hospitalized chronic heart failure patients [61, 62]. Hyperuricemia has a deleterious effect on the New York Heart Association (NYHA) class, exercise capacity, oxygen consumption, diastolic dysfunction, and cachexia [63–66]. Several longitudinal studies [67–70] and meta-analyses [71, 72] have assessed the association between UA and heart failure and found that elevated UA levels not only act as a risk factor for heart failure incidence, but are also associated with the severity of the disease and poor prognosis. Moreover, the Framingham Offspring Cohort Study reported that heart failure incidence rates were about sixfold higher among those at the highest quartile of serum UA (> 6.3 mg/dL) compared to those at the lowest quartile (< 3.4 mg/dL) and the adjusted hazard ratio was 2.1 (95% CI 1.04–4.22) [73]. A recent meta-analysis demonstrated that for every 1 mg/dL elevation in serum UA level, the odds of heart failure development increased by 19% (HR 1.19, 95% CI 1.17–1.21), and the risk of all-cause mortality increased by 4% (HR 1.04, 95% CI 1.02–1.06) [72]. Furthermore, in the British Regional Heart Study, treated hypertensive men with serum UA levels above 410 μmol/L showed an increased risk of heart failure of more than twofold compared to those on treatment with levels below 350 μmol/L, even after adjustment for confounding factors [74]. In addition, this study showed that serum UA may be a valuable prognostic marker for heart failure risk in older adults who were treated with hypertension. Although the mechanisms or pathways in which UA affects heart failure development have not yet been clearly identified, it has been postulated to be due to xanthine oxidase upregulation, renin-angiotensin-aldosterone system (RASS) activation, and use of diuretic drugs that may reduce UA excretion [75–78]. Based on these assumptions, serum UA lowering therapy with xanthine oxidase inhibitors such as allopurinol or febuxostat has shown clinical benefits in heart failure patients [79, 80]. However, some studies have not revealed noteworthy benefits of UA lowering therapy with xanthine oxidase inhibitors in heart failure patients with hyperuricemia; hence, further studies are needed [81, 82].

## Uric acid and atrial fibrillation

Recent studies have shown that elevated UA levels are associated with increased risk of atrial fibrillation. A European cohort study involving approximately 6000 patients showed that high baseline UA levels increased the risk of atrial fibrillation [60]. In a study on diabetic patients, the elevated UA levels were associated with the risk of atrial fibrillation [83]. Additionally, recent meta-analysis studies have shown that increased UA levels were associated with an increased risk of atrial fibrillation [84, 85]. This association was known to be greater in women than in men. The Atherosclerosis Risk in Communities (ARIC) study reported that elevated UA levels increased the risk of atrial fibrillation by 1.16 times, especially in women and blacks [86]. Moreover, our large cohort study has shown that elevated UA levels had a pronounced and independent association with the risk of atrial fibrillation, which was greater in women than in men [87]. Several studies have further suggested a link between sexual differences and UA levels because estradiol plays a role in protecting the endothelial cells and lowering the UA levels [88, 89]. The mechanism underlying the association between UA and the risk of atrial fibrillation has not been fully elucidated. However, it has been explained that UA causes atrial remodeling by inducing inflammation, oxidative stress, RASS activation and endothelial dysfunction, thereby increasing the risk of atrial fibrillation [90–92]. UA causes electrical remodeling, which shortens the atrial refractory period and establishes a reentry circuit in the atrium [91]. Furthermore, UA causes structural remodeling and slows the conduction velocity, thereby allowing reentry [90]. Elevated UA levels increase the nicotinamide adenine dinucleotide phosphate (NADPH) oxidase and xanthine oxidase activity, which in turn increase the reactive oxygen species. Such mechanisms may correspondingly contribute to the pathological consequences of atrial fibrillation such as thrombosis, inflammation, and tissue remodeling [93]. Based on these theories, a number of experimental studies have been published stating that inhibiting xanthine oxidase and NADPH oxidase reduces oxidative stress, and that N-acetylcysteine usage as an antioxidant may be beneficial in atrial fibrillation treatment or prevention. However, further research will be needed to determine the usefulness of these drugs, as research on humans, largely conducted based on highly defined policies, has raised controversial questions due to the disparate outcomes. Most of these studies have been conducted on a limited basis with highly defined populations.

## Uric acid and insulin resistance / metabolic syndrome

Metabolic syndrome (MetS), once called ‘Syndrome X’ by Raven in the year 1988, is a group of risk factors that increase CVD [94]. From that moment on, there has been some confusion in diagnosis due to the existence of various criteria that define MetS [95–97]. However, several organizations and expert groups, such as the National Cholesterol Education Program Adult Treatment Panel III (NCEP:ATPIII), American Association of Clinical Endocrinologists (AACE), International Diabetes Federation (IDF), and American Heart Association/National Heart, Lung, and Blood Institute (AHA/NHLBI), continue to attempt to incorporate different parameters for diagnosing MetS [98]. The NCEP:ATPIII defined the components of MetS related to CVD as abdominal obesity, atherogenic dyslipidemia, elevated blood pressure, insulin resistance, and pro-inflammatory and pro-thrombotic states [99]. Insulin resistance refers to a pathological condition that results in abnormally low insulin sensitivity at the physiological insulin levels, which eventually leads to hyperinsulinemia [100]. Historically, hyperuricemia was frequently observed in MetS, but this was thought to be due to the secondary effect of hyperinsulinemia due to the decreased renal excretion of UA by distal tubular reabsorption [101]. However, a recent epidemiologic study conducted by our group has shown that hyperuricemia often precedes the development of insulin resistance, and that serum UA is an independent risk factor for MetS, including insulin resistance [23]. Additionally, xanthine oxidase inhibitors such as allopurinol not only decreased the UA levels, but also improved insulin resistance and systemic inflammation in asymptomatic individuals with hyperuricemia [102]. The precise mechanism of UA-induced insulin resistance is not yet clear; however, two hypotheses have been suggested. The first hypothesis is that UA inhibits insulin-induced endothelial nitric oxide synthase (eNOS) phosphorylation and subsequent nitric oxide (NO) production, thereby contributing to insulin resistance; therefore, using UA lowering agents such as allopurinol improves insulin resistance [103]. The second hypothesis is that UA affects adipocytes by upregulating the pro-inflammatory factors and downregulating the insulin sensitizers and anti-inflammatory factors [104]. Experiments using mouse models of MetS have shown that lowering UA by xanthine oxidase inhibitors in obese mice with MetS can improve the inflammatory endocrine imbalance in adipose tissue by increasing the production of adiponectin [104]. Based on these studies, it is necessary to clarify the mechanism by which UA causes insulin resistance in humans and conduct large-scale clinical studies to determine the effect of lowering UA levels on insulin resistance.

## Uric acid and non-alcoholic fatty liver disease

Non-alcoholic fatty liver disease (NAFLD) refers to the condition of fat infiltration in the hepatic parenchyma without alcohol abuse, which can potentially lead to liver cirrhosis or liver cancer, and is known to be associated with coexisting conditions such as obesity, type 2 diabetes mellitus, and hyperlipidemia [105]. Recently, a growing number of studies, including our study, have suggested that the elevated serum UA level is associated with an increased risk of NAFLD [24, 106]. The association between serum UA and NAFLD was greater in the obese population as well as in women than in men [107]. In addition, experimental studies using animal models have demonstrated that UA lowering therapy may help in the development and treatment of NAFLD [108, 109]. The mechanism involved in NAFLD development by UA is not yet clear; however, several hypotheses have been suggested. The first is that increased UA levels increase reactive oxygen species (ROS) production and oxidative stress, leading to pro-inflammatory endocrine imbalance [104]. Second, UA induces lipogenesis by endoplasmic reticulum generation and activation of fatty acid synthase and acetyl-CoA carboxylase, thereby leading to fat accumulation in the hepatocytes [110]. Lastly, UA deteriorates the endothelial function and nitric oxide bioavailability causing insulin resistance, which is the most significant mechanism, resulting in hyperinsulinemia [108, 111].

## Conclusions

As gout prevalence has increased over the past decades, there has been a growing interest in UA, the causative agent of gout. Although there are some controversies due to the ambivalent nature of UA in the human body, several clinical and experimental studies have shown that UA is associated with CVDs and MetS. Moreover, a number of studies have revealed that UA is an independent risk factor for these diseases, suggesting that UA may be a potential therapeutic target for cardiometabolic disease patients, especially those with hyperuricemia, as UA plays a central role. But, there has been some controversy about the causal relationship between hyperuricemia and cardiometabolic disease; uncertainties exist regarding the mechanism of UA-induced cardiometabolic disease. Therefore, it is necessary to elucidate the causal relationship between UA and cardiometabolic disease by further well-controlled, large-scale studies as well as reveal the potential therapeutic and preventive effects of UA lowering therapy in cardiometabolic disease.

## Data Availability

Not applicable.

## Abbreviations

UA: Uric acid
NAFLD: Non-alcoholic fatty liver disease
CVD: Cardiovascular disease
CHD: Coronary heart disease
MI: Myocardial infarction
RASS: Renin-angiotensin-aldosterone system
MetS: Metabolic syndrome
